# Efficacy of Revolution®Plus in cats for the prevention of *Dipylidium caninum* transmission by infected *Ctenocephalides felis*

**DOI:** 10.1186/s13071-025-06950-5

**Published:** 2025-08-04

**Authors:** Lindsay Weaver, Alta Viljoen, Riaan Maree, Lina D’Hanis, Shelby Jones, Jane Tonso, Reinier Zweiger, Julian Liebenberg, Vickie L. King, Keith Baker, Jessica Rodriguez, Chris Adolph, Thomas Geurden

**Affiliations:** 1 Zoetis, Veterinary Medicine Research and Development, 333 Portage St, Kalamazoo, MI 49007 USA; 2 Clinvet, South Africa Uitzich Road, Bainsvlei, Bloemfontein, 9338 South Africa; 3 Clinvet, 1479 Talmadge Hill Road South, Waverly, NY 14892 USA; 4 Zoetis, Veterinary Medicine Research and Development, Mercuriusstraat 20, 1930 Zaventem, Brussels Belgium; 5 Q-Vative, 15 Willowood, Kamoa Cresent, Wild Olive Estate, Bloemfontein, 9301 South Africa; 6 Zoetis 10 Sylvan Way, Parsippany, NJ 07054 USA

**Keywords:** Cat, Cestode, *Ctenocephalides felis*, *Dipylidium caninum*, Flea, Revolution^®^ plus, Selamectin, Sarolaner

## Abstract

**Background:**

Revolution^®^ Plus is a topical combination drug product containing selamectin and sarolaner that has been proven effective against the cat flea *Ctenocephalides felis*, the intermediate host of the cestode *Dipylidium caninum*. Here, we report two studies evaluating the efficacy of a single administration of Revolution Plus in preventing *D. caninum* infection in cats for 1 month through killing of the flea intermediate host.

**Methods:**

Two studies (study 1 and 2) with the same design were conducted. In both studies, 2 treatment groups of ten cats each were enrolled. On Day 0, the cats in group 1 were treated with a placebo, and the cats in group 2 were treated with Revolution Plus at the minimum recommended dose of 6.0 mg/kg selamectin and 1.0 mg/kg sarolaner. After treatment on Day 0, as well as on Days 7, 14, 21, and 30, the cats in both treatment groups were infested with 100 (± 5) unfed, *D. caninum*-infected fleas. Live flea counts were conducted on Day 33 (72 ± 2 h after Day 30 infestation). All cats were euthanized on Day 58, and necropsies were performed to enumerate *D. caninum* scolices in the gastrointestinal tract.

**Results:**

In both study 1 and 2, all placebo-treated cats were infested with two or more *D. caninum* scolices at necropsy. Significantly lower mean flea counts were recorded for the Revolution Plus-treated cats compared with placebo-treated cats (*P* ≤ 0.0001), and efficacy based on least squares mean flea counts on Day 33 was 100% (in study 1) and 94.3% (in study 2). Scolex counts were also significantly decreased in Revolution Plus-treated cats compared with placebo-treated cats, with a 97.1% efficacy in study 1 and a 99.3% efficacy in study 2.

**Conclusions:**

One topical administration of Revolution Plus at the minimum dosage of 6.0 mg/kg selamectin and 1.0 mg/kg sarolaner provided high efficacy in the prevention of *D. caninum* infection through the killing of its vector, *C. felis*, for an entire month.

**Graphical Abstract:**

**Figure:**
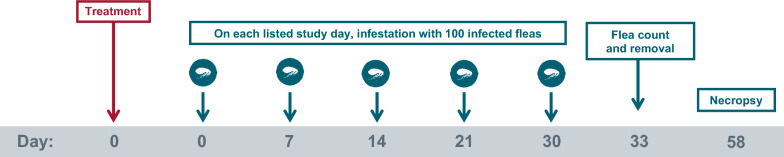

## Background

*Dipylidium caninum* is found worldwide in many vertebrate hosts, including canids and felids [1]. The cestode has an indirect lifecycle, requiring an intermediate flea or, less commonly, a louse host. The cat flea, *Ctenocephalides felis*, is considered the most common intermediate host due to its widespread distribution and consistently high prevalence on cats and dogs globally [2–6]. In the vertebrate host, adult tapeworms reside in the intestines and release egg packets or proglottids that are passed into the environment via the stool. As proglottids break down in the environment, eggs are released and then ingested by flea larvae, and they further develop into the infective cysticercoid stage [7]. Transmission of *D. caninum* to cats occurs through the ingestion of *C. felis* adults containing cysticercoids, with the habitual behavior of regular and thorough grooming thought to contribute to the risk of exposure to infected fleas [1, 8]. Infection does not typically induce symptoms, although general clinical signs associated with parasitosis may be observed, such as anal pruritus associated with proglottid migration, diarrhea, weight loss, and poor hair coat [1].

The low host specificity of *C. felis* is a major factor in its widespread occurrence, and the flea species has been reported from a wide variety of hosts, including feral cats and wildlife [9]. Flea-infested cats contribute to the continuous deposition into the environment of flea eggs, which develop into adult fleas that are readily picked up by cats that interact with these outdoor areas [10]. Although humans are not common hosts of *D. caninum*, infants and children can be especially vulnerable to infection due to their close contact with animals and their less rigorous hygiene habits. As with cats, human infections are largely asymptomatic, although infections may induce abdominal pain, diarrhea, and anal itching [1, 11–13].

The preferred treatment for *D. caninum* in cats is praziquantel or epsiprantel; however, recent reports of sporadic resistance developing in *D. caninum* against praziquantel have been noted [14–16]. Additionally, neither of these anthelmintics addresses the problem of environmental contamination with *D. caninum*-infected *C. felis* or the subsequent risk of reinfection and the need for additional endoparasiticide treatment. Ectoparasiticides can play a critical role in the fight against flea-borne diseases, acting rapidly against infestations on the host and effectively eliminating the need for environmental treatment [17]. Revolution Plus^®^ (Zoetis; marketed as Stronghold Plus^®^ in Europe) is a spot-on formulation for cats combining selamectin and sarolaner, and was shown to be well-tolerated, safe, and effective against infestations with *C. felis* [18–21]. Here, we describe the efficacy of Revolution Plus in preventing *D. caninum* infection in cats for 1 month through killing of the flea intermediate host, *C. felis*, when administered at a minimum dosage of 6.0 mg/kg selamectin and 1.0 mg/kg sarolaner.

## Methods

These placebo-controlled laboratory comparative efficacy studies were conducted by ClinVet in Bloemfontein, South Africa (study 1), and New York, USA (study 2). All cats were under the care of a licensed veterinarian at all times, and studies complied with all applicable local laws, state laws, and national regulations related to the humane care and use of animals. All study protocols were approved by the Study Site Institutional Animal Care and Use Committee, and all study procedures were in accordance with the relevant World Association for the Advancement of Veterinary Parasitology guidelines [22–24].

### Study design

**Fig. 1:**
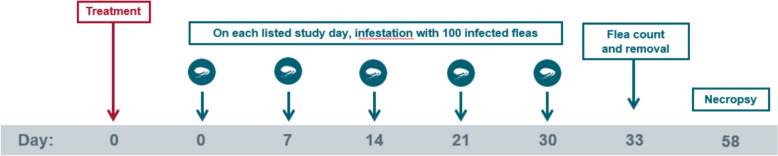
Study design used in study 1 and 2

The study design used in both study 1 and 2 is provided in Fig. 1. The host suitability for *C. felis* was determined by infesting candidate cats (*n* = 24 per study) on Day −8 (study 1) or Day −21 (study 2) with *C. felis* (not infected with *D. caninum*). At 24 h post-infestation, cats were examined and comb-counted, and the 20 cats with the highest flea counts were selected for each study. Cats were allocated to treatments, rooms, and cages according to a randomized complete block design with blocking based on pre-infestation flea counts. Blocks consisted of two cats, with one cat randomized to each treatment within the block. Cats in the same block were randomized to cages located near each other within each room. Enrolled cats were moved to their allocated cages within 8 days of study start and treated on Day 0 with either placebo or with Revolution Plus. All cats were infested with 100 (± 5) viable, unfed, adult *C. felis* fleas on Days 0, 7, 14, 21, and 30. Cats were allowed to engage in normal grooming behavior throughout the course of the study to mimic the natural infection method of *D. caninum*. Live flea counts were conducted on Day 33, with removal of the fleas. All cats were euthanized on Day 58, and necropsies were performed to recover, identify, and count *D. caninum* scolices from the gastrointestinal tracts of each cat. Masking was accomplished by the separation of the functions of study personnel. All persons making observations, performing polymerase chain reaction (PCR) testing; conducting scolex counts, post-treatment flea infestations, and post-treatment flea counts; or performing general care for the cats were masked to experimental treatments.

### Animals

All cats enrolled in these two studies were healthy and clinically normal as determined by a veterinarian with experience in the practice of general feline medicine. All enrolled cats were confirmed negative for *D. caninum* infections based on PCR analysis of fecal samples [25] within 14 days of study start on Day 0. Study 1 utilized 12 males and 8 females aged 16–113 months and weighing 2.8–4.7 kg on Day −1. Study 2 utilized ten males and ten females aged 8–47 months of age and weighing 2.7–7.7 kg on Day −1. Cats were intact or neutered, none were pregnant or lactating, and all were uniquely identified. All cats had been regularly dewormed and previously vaccinated as required, but no cat was given medication from Day −14 (study 1) or Day −21 (study 2) through study end (unless concomitant treatment was required to maintain adequate care). All cats were housed in individual, walled cages such that no physical contact between cats or transmission of fleas was possible, and cats were not moved between cages after Day 0. Cats were fed a commercial dry food once per day, and fresh water was provided ad libitum. General health observations were performed daily for each cat starting on Day −14 (study 1) or Day −21 (study 2) through the duration of the study.

### Flea infestations and assessment

Study 1 used viable, unfed adult *C. felis* fleas from a colony originally sourced from PLRS laboratories (Corapeake, North Carolina, USA) in 2010 and enriched with fleas from Sierra Research Laboratories (Modesto, CA, USA) in 2017. Study 2 used viable, unfed adult *C. felis* originally sourced from Ecto Services, Inc. (Henderson, North Carolina, USA) in 2020 and enriched with fleas from Henderson, North Carolina, in 2022. All fleas were infected for the purpose of the studies with *D. caninum*, collected from cats in the field either in Thessaloniki, Greece, in 2019 (study 1) or in Oklahoma, USA, in 2015 (study 2), as follows: freshly collected flea eggs were added to *D. caninum* proglottids for a period of 3 days. After the flea eggs hatch, the resulting larvae are exposed to the proglottids and are thereby forced to feed on them. These flea larvae were then transferred to a standard flea breeding medium, where they were allowed to complete their developmental cycle. The fleas were confirmed to be infected with *D. caninum* at an infection rate ranging from 23.0% to 53.3% in study 1 and from 6.8% to 20.0% in study 2. Both in study 1 and 2, a total of 24 cats were infested with 100 (± 5) viable, unfed adult (and *D. caninum*-free) *C. felis* fleas to determine host suitability. Then, 1 day after infestation, the number of live fleas present on each cat was counted, and fleas were removed, and the 20 cats with the highest flea counts were randomly allocated to one of two treatment groups blocked on flea counts. After treatment, cats were infested with 100 (± 5) viable, unfed adult and infected fleas on Days 0, 7, 14, 21, and 30. During infestations, cats remained in their respective cages. A vial containing 100 (± 5) fleas was swirled to form a flea pellet, which was then deposited onto the cat’s hair coat as close as possible to the skin at a site distal to the site of treatment. The vial was held in position for ~10 s to ensure the flea pellet was not dislodged and to facilitate dispersal of the fleas into the hair coat.

Live flea counts with removal of the fleas were conducted on Day 33, 72 (± 2) h after Day 30 infestation. Each cat was removed from its cage and placed upon a disinfected table. Fine-toothed flea combs were used to comb the entire body of the cat, starting with the anterior and working toward the posterior end and ventral areas. Any collected fur was removed from the comb and separated, and fleas within the fur were counted. Cats were combed for at least 10 min. Personnel conducting counts were masked to treatment assignments and changed gloves between each cat.

All cats were euthanized on Day 58. The gastrointestinal tracts were removed and ligated to separate the stomach, small intestines, large intestines, and the end of the rectum. Each section was cut open, and the mucosa was observed macroscopically for the presence of worms, which were collected and preserved. The mucosa was washed with water and scraped, with the washings and scrapings collected separately, washed through 0.150 mm sieves. The collected material was examined using a stereomicroscope, and the number of scolices were counted and recorded.

### Treatment and observations

Clinical observations and administration site evaluations were performed on all cats prior to treatment on Day 0. All cats were then treated with either placebo (Revolution Plus formulation without the active ingredients) or with Revolution Plus at its minimum recommended dose (6.0 mg/kg selamectin, 1.0 mg/kg sarolaner). Doses were calculated using body weights recorded on Day −1. The entire dose was administered to the skin at a single site, at the base of the neck cranial to the scapulae. The site of administration was evaluated at 1 h (± 15 min), 3 h (± 30 min), 6 (± 1) h, and 24 (± 1) h after treatment as well as on Days 3, 5, and 58 for cosmetic changes (matting, spiking/stiff hair, wetness, white deposits), alopecia, erythema, and edema. Clinical observations were completed at 1 h (± 15 min), 3 h (± 30 min), 6 (± 1) h, and 24 (± 1) h after treatment.

### Statistical analyses

Adequate challenge was defined as 60% of the placebo-treated control cats maintaining ≥ 50 fleas following the final flea infestation on Day 30 and 60% of the placebo-treated control cats having ≥ 2 adult *D. caninum* at the time of necropsy. The experimental unit was the individual cat. Scolex counts and flea counts were analyzed with a general linear mixed model containing the fixed effect of treatment and the random effects of room. For both scolex and flea counts, the treatments were compared, and efficacy was calculated as follows: 100 × (control mean – revolution plus mean)/control mean. For flea counts, means, standard errors, 95% confidence limits, minimums, and maximums were calculated. Efficacy was calculated using the least squares means as estimates of the arithmetic means. Scolex counts were transformed with a natural logarithm transformation prior to analysis. Least squares means, standard errors, 95% confidence limits, minimums, and maximums were calculated. Efficacy was calculated using the geometric (back-transformed least squares) means.

## Results

### Dosing and observations

All cats in both studies received a complete dose of either placebo or Revolution Plus. No cats in either study were observed to have discomfort at the site of administration. No cats were reported to have post-treatment clinical observations. For study 1, two cats in the placebo-treated control group were noted prior to treatment to have pre-existing mild alopecia that resolved within 5 days without concomitant treatment. A third cat in the placebo-treated group was noted on Day 12 to have a blocked nose, which resolved by Day 21 following treatment. No abnormal health events were observed, and no concomitant medications were administered during study 2.

### Flea counts

Day 33 flea counts revealed that placebo-treated control cats had adequate infestations, with 90% of placebo-treated cats in study 1 and 60% of placebo-treated cats in study 2 having ≥ 50 fleas following the final flea infestation on Day 30 (Table 1). On that day, flea counts in the placebo-treated control group ranged from 44 to 176 in study 1 and from 22 to 102 in study 2. All Revolution Plus-treated cats in study 1 and nine of the ten Revolution Plus-treated cats in study 2 were free of fleas on Day 33, and there was a statistically significant decrease in mean flea counts from Revolution Plus-treated cats compared with placebo-treated cats (*P* ≤ 0.0001) in both studies. Efficacy based on least squares mean flea counts was ≥ 94.3% in both studies. Least squares means were used as estimates of the arithmetic means.

**Table 1:** *Ctenocephalides felis* counts for cats treated with placebo or Revolution® Plus and percent efficacy

Study	Treatment^a^	Cats infested^b^ (*n*)	Arithmetic mean	Range	Geometric mean	Least squares mean^c^	Standard error	95% confidence limits	Efficacy^d^ (%)	*P*-value^e^
1	Placebo	10	96.2	44–176	89.8	96.2	8.3	77.4–115.0	—	< 0.0001
	Revolution Plus	0	0.0	0–0	0.0	0.0	8.3	−18.8 to 18.8	100	
2	Placebo	10	56.3	22–102	51.7	56.3	7.6	39.2–73.4	—	0.0001
	Revolution Plus	1	3.2	0–32	0.4	3.2	3.2	−4.0 to 10.4	94.3	

^a^*n* = 10 per group

^b^Nine (study 1) and six (study 2) cats in the placebo-treated control group had ≥ 50 fleas

^c^Least squares means are used as estimates of the arithmetic means

^d^Efficacy based on least squares means relative to placebo on Day 33

^e^Significant difference (*P* ≤ 0.05) between treatment groups

### Scolex counts

Scolex counts showed 80% (study 1) and 100% (study 2) of placebo-treated control cats had ≥ 2 *D. caninum* scolices, indicating adequate infections in both studies. Scolex counts in *D. caninum*-infected control cats ranged from 0–157 in study 1 and 40–255 in study 2 (Table 2). In contrast, only one Revolution Plus-treated cat in study 1 and two Revolution Plus-treated cats in study 2 were infected with *D. caninum*, with the highest recorded scolex count being 29. In both studies, scolex counts in Revolution Plus-treated cats were significantly decreased compared with placebo-treated cats (*P* ≤ 0.0046; Table 2), and the efficacy of Revolution Plus based on least squares mean scolex counts in both studies was ≥ 97.1%.

**Table 2:** *Dipylidium caninum* counts for cats treated with placebo or Revolution® Plus and percent efficacy

Study	Treatment^a^	Number of cats infected^b^ (*n*)	Geometric mean	Arithmetic mean	Range	Back transformed standard error	Back transformed 95% confidence limits	Efficacy^c^ (%)	*P*-value^d^
1	Placebo	8	12.9	34.2	0–157	5.9	4.2–36.2	—	0.0046
	Revolution Plus	1	0.4	2.2	0–22	0.2	−0.5 to 2.7	97.1	
2	Placebo	10	93.2	106.5	40–255	15.8	63.4–136.8	—	< 0.0001
	Revolution Plus	2	0.7	3.4	0–29	0.3	−0.3 to 2.9	99.3	

^a^*n* = 10 per group

^b^Infection = all positive cats in the placebo-treated cats had ≥ 2 scolexes identified during necropsy on Day 58

^c^Efficacy based on least squares means relative to placebo

^d^Significant difference (*P* ≤ 0.05) between treatment groups

## Discussion

Treatment with Revolution Plus significantly lowers mean live flea counts compared with placebo-treated cats within 12 h of treatment, persistently controls flea infestations for at least 5 weeks, and reduces flea egg production by ≥ 99.9% for up to 5 weeks [18–21]. In both current studies, a single dose of Revolution Plus was effective (≥ 94.3%) in treating *C. felis* infestations of cats, and Revolution Plus-treated cats had significantly reduced mean flea counts compared with placebo-treated cats, with 19 of the 20 Revolution Plus-treated cats free of fleas on Day 33.

A fast and persistent efficacy against ectoparasites is important in reducing the risk of pathogen transmission to hosts [26–29], as well to prevent the continuous deposition of flea eggs into the environment. Cats are known to ingest up to 17.6% of their fleas each day during grooming [30], which can facilitate the transmission of *D. caninum*. The primary aim of these two studies was to evaluate whether the efficacy of Revolution Plus against *C. felis* would provide cats with protection from *D. caninum* infection. All placebo-treated cats in both studies had *D. caninum* infections in most of these cats (80% in study 1; 100% in study 2). In contrast, only three Revolution Plus-treated cats across both studies were infected with *D. caninum*, and scolex counts in Revolution Plus-treated cats were significantly decreased compared with placebo-treated cats. The efficacy of Revolution Plus in preventing *D. caninum* infection was ≥ 97.1% in study 1 and 99.3% in study 2, providing evidence that a single treatment of Revolution Plus at the minimum effective dose protects cats for the entire month from infection with *D. caninum* through killing of its vector *C. felis*, and can aid in the prevention and management of *D. caninum* infections.

## Conclusions

A single topical administration of Revolution Plus at the minimum dosage of 6.0 mg/kg selamectin and 1.0 mg/kg sarolaner provided high efficacy against *D. caninum* infection in cats through the killing of its vector, *C. felis*, for an entire month.

## Data Availability

The dataset supporting the conclusions of this article is included within the article.

## Abbreviations

PCR: Polymerase chain reaction
